# Incorporating the Co-Creation Method into Social Innovation Design to Promote Intergenerational Integration: A Case Study of a Public Square

**DOI:** 10.3390/ijerph191912908

**Published:** 2022-10-09

**Authors:** Jianbin Wu, Linghao Zhang, Xiangfang Ren

**Affiliations:** School of Design, Jiangnan University, Wuxi 214000, China

**Keywords:** community mental health, co-creation design, intergenerational integration, social innovation, design method, design strategy

## Abstract

With the COVID-19 pandemic sweeping the world, there is an increased focus on intergenerational relationships, community mental health issues, and well-being in community contexts. This paper assesses the effectiveness of a co-creation approach for intergenerational integration at the theoretical level. The study used a collaborative co-creation design method in the community design process to explore whether the participation of community residents of all ages in addressing community issues promoted communication and alleviated stereotyping between the various generations. This study was conducted in Shanghai’s Hongqiao New Village square, where we conducted participatory research and co-creation workshops in response to the demand for the use of the public space as a location for social interactions. The results showed that intervention in community creation through collaborative design is conducive to promoting friendly relations among different age groups, forming a sense of social security and thus enhancing social well-being. Finally, this paper combines practical cases and theoretical models of collaborative co-creative design approaches to promote the intergenerational integration of communities and is summarized from the input and output parts as well as the influencing factors and constraints of the collaborative co-creative. In addition, it provides new ideas on how to improve intergenerational relationships and form a positive and sustainable community mental health environment in the future.

## 1. Introduction

Two common goals for community development come from broad group participation and effective problem solving. Research has shown that groups with positive images, positive experiences and positive attributions in social life also show higher self-esteem, life satisfaction and happiness, and they are more likely to be involved in solving community problems. People with negative perceptions of the future, social experiences and attributions are also prone to depression, dysfunction, helplessness and negative effects [1]. This shows that the relationship between cultivation and physical and mental guidance for residents is an important part of the development process in creating communities. Therefore, in communities composed of different generational age groups, maintaining friendly community relations and guiding residents toward positive physical and mental health are important trends being explored today.

Several pressing societal issues, including preventing social isolation and combatting the problems associated with aging populations, can also be addressed through group collaboration. Many initiatives, including the ‘All Age Society’ proposal of the World Assembly on Aging and the activities associated with the European Year of Active Ageing and Intergenerational Solidarity demonstrate an awareness of the unaddressed problems associated with aging populations [2]. In this context, the development of intergenerational practices becomes a mechanism through which to enhance intergenerational proximity, improve intergenerational understanding and communication, and foster a commitment to reciprocity and solidarity [3]. As a result, discussions about intergenerational issues during COVID-19 have been increasingly focused on and reshaped by the concepts of integration and responsibility amongst the generations [4].

Research theories related to intergenerational issues mainly focus on the fields of social psychology, mental health, sociology and communication, which are disciplines that stress instinctive communication [5,6], although the promotion of intergenerational identity requires a multidimensional form of integration, especially for psychological and cultural integration [7]. However, there is little systematic discussion of how to intervene in community creation through a complete design methodology for the sustainable dynamic development of community in the future.

Meanwhile, this paper is an in-depth exploration of the phenomenon of intergenerational disconnects, conflicts, and stereotypes by presenting them as a focus of community public health research. Health is a relatively broad concept. In this paper, we focus on community mental health because the literature and practice have shown that excessive intergenerational disconnection and conflict have a negative impact on group mental health. Positive intergenerational interactions (communication, social engagement) can contribute positively to group mental health [8,9,10].

Therefore, how to improve the quality of intergenerational interactions through the intervention of design methods and theories, and ultimately form a positive mental health environment for the masses? This paper is a preliminary exploration of these questions.

In addition, co-creation design approaches are becoming increasingly popular in the field of social design. These approaches are used to investigate, generate and develop new ways to achieve collective social goals [11,12]. Co-design is based on concept of ‘radical democracy’ [13], which focuses on the conflicting needs and interests of the public by synergizing and integrating concepts in an open space.

As such, this study intervenes in the overall process of community design by adopting a collaborative co-creation approach, using a public square in a Shanghai community as a practical model to investigate whether residents can solve problems, mitigate negative intergenerational emotions and improve intergroup relations by talking and collaborating with each other, thus modeling a co-creation design approach for intergenerational community development.

## 2. Literature Review

We analyzed current trends from the multidisciplinary perspectives of design, sociology, semiotics and psychology, and we systematically explored the ways that intergenerational groups have built trusting relationships and constructed intergenerational cognition [14].

### 2.1. Community Mental Health, Intergenerational Integration and Co-Creation Theory

The American Psychological Association defines community mental health as activities that promote mental health in the community rather than in institutional centers [15]. The main focuses of community mental health services are treatment, mitigation, relapse avoidance, primary prevention and health promotion. The common denominator in these directions is the focus on the complementary role of cultural (cross-cultural, etc.) elements in the community framework. As a result, concepts such as community mental health care [16] and community mental health team management were born [17]. This shows that community diversity activities are effective in bringing residents together and mitigating negative emotions in order to maintain positive and healthy community development.

Furthermore, what is the definition of intergenerational integration? The concept has two distinct but interconnected meanings. The first relates to breaking through age barriers and stipulates that a person’s age does not determine their societal role. The second relates to cross-age interactions, or the co-participation of different age groups in collective activities [18]. Both meanings stress the importance of intergenerational integration for both older and younger individuals. Intergenerational integration (I-I) enables older individuals to enhance their well-being by participating more fully in society. These individuals can contribute their experience, wisdom and expertise, and receive respect from younger people for their contributions to society, thus breaking down age boundaries. Hagestad noted that when young people understand the importance of giving to others, society develops in a positive and sustainable direction by, for example, providing services for older adults [19]. In addition, the simple act of providing a space for older people to share their experiences with younger people can promote mutual understanding and reduce discrimination.

Co-creation is an active, creative and social process that depends on the collaboration of organizations and participants. Co-creation provides opportunities for all involved and creates value for stakeholders [20,21,22]. The practice is centered on the process rather than the result, and it can thus be used to build bridges for research teams that facilitate participant integration and communication, which provides further creative inspiration.

In addition, co-creation’s contribution to the I-I of communities is multifaceted. Concepts such as the link between democratic innovation and individualism are key in promoting community co-creation. In communication studies, for example, adjustment strategies such as integration and divergence are characterized by social convergence (collectivism) rather than by individuality (individualism). Although the two adjustment strategies focus on different elements, they both serve as effective approaches to fostering interpersonal relationships. According to Calvo and De Rosa, ‘competitivism’ was a model of democracy in which individuals were placed in a competitive space and driven by varying motivations to express and direct themselves collectively and positively through interpersonal confrontation [23]. In this way, I-I is part of a dynamic process whereby the value of collective wisdom is maximized, thus contributing to stable societal development.

### 2.2. The Relationship between Community Mental Health, Intergenerational Integration and Co-Creation Theory

Firstly, intergenerational relationships are an important part of mental health improvement, and we can achieve that by promoting intergenerational relationships that benefit community mental health. The community is also experimenting with multiple approaches to research, such as Biro-Hannah’s use of art to mitigate the psychological effects of the body as a way to effectively manage multiple mental health and emotional problems [24]; Heather Gridley and others organize community arts choruses to promote public cohesion among community residents and enhance their self-confidence, empowerment, well-being and interpersonal skills [25]. In addition, in the current environment, the new crown epidemic has changed the path of our lives and has also affected our mental health. Loss of livelihoods and loved ones, social exclusion, and intergenerational tensions are more prominent in the crisis [26]. Therefore, it is all the more important for us to take active measures to meet critical tests and revitalize our communities.

**Figure 1:**
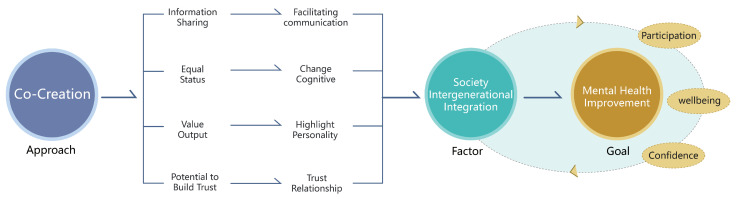
The Connectedness of Community Mental Health, Intergenerational Integration and Co-Creation Theory.

Secondly, the goal of intergenerational integration is complex and includes a variety of elements. These include promoting equal rights for all generations, emphasizing the unique characteristics of each group, encouraging the various generations to communicate with and learn from each other, cultivating a high sense of community belonging and encouraging individuals to participate in the creation and development of the community. As such, the process of promoting I-I in communities can be further subdivided into four steps: facilitating communication, improving cognition, expressing individuality and building trust relationships [27]. The direct correlation between collaborative co-creation and social intergenerational integration led us to the following research hypothesis: community groups of different ages facilitate intergenerational integration through mutual communication and collaboration in a cycle of collective activity [28,29]. To verify this hypothesis, we used an in-depth verification process for our findings (Figure 1).

Finally, we can see from the figure that the three are in a cascading relationship. By promoting intergenerational integration through collaborative co-creation, we can improve the mental health problems of community residents in order to form a positive community environment.

## 3. Methods and Experiments

We proposed the theoretical validity of using co-creation to intervene in community design as a means of promoting community intergeneration [30,31]. To further test the validity of the theory, we carried out a practical project in a community in Shanghai, China, in which we assessed the existing situation and the problems of residents across all age groups regarding the use of a public plaza.

Intergenerational behavior in public spaces has been discussed globally in some depth. Many scholars have pointed out that an overemphasis on age divisions and age-specific institutions has led to increasing spatial and cultural distances between groups. This can lead to increased ageism and other social problems associated with age [32]. To weaken the intergenerational problems in the public space environment, scholars actively explore the positive development of group relationships from the concepts of self-compassion, social security, and grassroots innovation to generate a sense of psychological security through multiple experiences of mutual care, support, collaboration, and comfort, which in turn stimulates group innovation and enhances well-being [33,34,35,36].

Through the collaborative participation of the community members, we integrated the concept of co-creation design into the overall research process to discover the essential reasons for the emergence of conflicts in groups and generate effective ideas for the use of the community square. We also verified whether the process had reduced stereotyping in the community by the end of the experiment.

### 3.1. Project Background

The project began with a conflict in a neighborhood square. From 7 pm to 9 pm, the square was in peak use, featuring a square dance senior group (elderly dancers) as well as children and older people conversing. The disorderly trajectory of children moving in and out of the dance area led to frequent conflicts between the dancers, the children, and their parents. Children and dancers often collided physically with each other. Dancers would prohibit children from moving around the public space to avoid the potential for such collisions. The parents would then argue with the dancers that it was a public space, and their children had a right to use it. Neither side was willing to give way. In the long run, intergenerational relationships in the community have gradually deteriorated and have had a degree of negative impact on each other. As a result, few people participated in community activities. These problems had negative effects on neighborhood relations and on the friendly and sustainable development of the community.

The contradictory understandings of the purpose of the public plaza reflected the diverse needs of the community residents who used it. In response to the issues raised by the community members, we devised the following questions: (1) How can researchers comprehensively ascertain the thoughts and feelings of a group on a particular subject? and (2) What methods can be used to provide residents with a platform to express their views and cultivate a sense of belonging and respect across the community? We began our experiments based on the above questions.

### 3.2. Project Process

#### 3.2.1. Input: Participatory Research Activity

We used the participatory research method to explore the use of the plaza by community residents. Considering that communal activities took place mainly on weekends, we introduced the concept of community days by conducting multiple activities designed to encourage groups to interact. During the initial research phase, one-on-one interviews with semi-structured roles (researcher and respondent) were changed to a role-free approach using research cards, which was gamified to improve the participation of community residents. This modification allowed the participants to freely choose the discussion topics in which they were interested, thereby enabling them to articulate their true needs.

With the theme of ‘Community Truth or Dare’, we set up questions relating to the theme of the public square, ranging from the dimensions of the plaza to intergenerational relationships and unrestrained thinking. The research questions were based on the characteristics of the local community environment and combined the Mental Health Scale and the Intergenerational Attitude Scale to explore the real demands of the community residents. Furthermore, we need to understand the mental health of the local residents, thus laying the foundation for the subsequent design of the intervention [37,38]. The Mental Health Scale focused on “intergenerational tension and sensitivity”, “psychological imbalance” and “hostility” factors [39].

These questions were presented in the form of cards and combined with more practical issues of community spaces such as plazas, pavilions and fitness areas, to improve their readability. Participants answered the questionnaire according to its content or followed the instructions to complete the corresponding tasks. Examples include high-fiving strangers in the square, taking pictures together, playing games, etc. In addition, the cards featured a plaza map to orientate community members in the plaza space.

**Figure 2:**
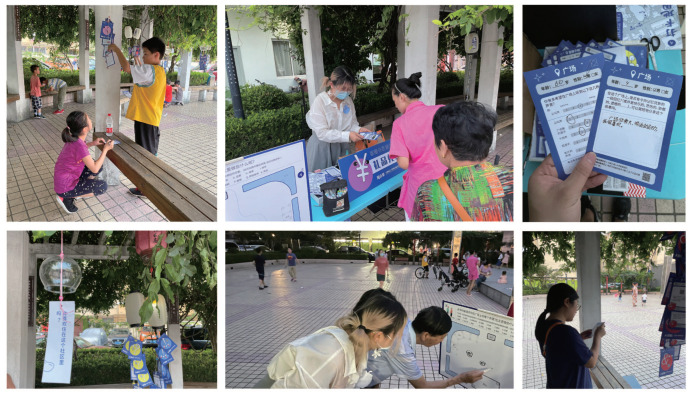
Community participatory research site.

A total of 30 local residents participated in the project. To ensure a relatively balanced number of participants in each age group, there were 10 children (10–18 years), 6 young-aged (18–35 years) (Y), 8 middle-aged (35–59 years) (M), and 6 older (59 years above) (O) participants. This categorization aided us in the interview statistics analysis and data analysis phases (Figure 2).

#### 3.2.2. Output: Co-Creation Workshop

We held a co-creation workshop and invited 30 residents who had participated in the participatory research activity to complete the co-creation activities. The theme of this workshop was “Build our own community square”, in which participants collaborated, discussed and designed their own ideal community public space (concept plan). The purpose of the workshop was to understand local residents’ vision of the future of community space and to provide solutions to the problems of daily community square space. It was an attempt to build an effective communication platform to encourage participants from all generations to express their true thoughts. Through collaborative discussions, participants would be able to promote mutual awareness and help alleviate stereotypes.

The workshop process featured topics that elaborate on impressions of the community as a way to trigger empathy between groups. We set up intergenerational collaboration sessions and arranged a Professional Mental Health Facilitator. On the one hand, they solved the psychological confusion of the participants. On the other hand, they improved the quality and efficiency of intergenerational communication in the co-creation process.

In the design of the toolkits, we used Geographical Information Systems (GIS) to accurately create a spatial map of the local community, which was used as the basis for the co-creation workshop [40,41]. In addition, to ensure that participants of all ages can identify spatial orientation and recognize each location, we tried to recreate the situation as much as possible from the visual aspect. We also introduced the map to the residents in a comprehensive manner during the workshop so that participants could develop spatial awareness.

**Figure 3:**
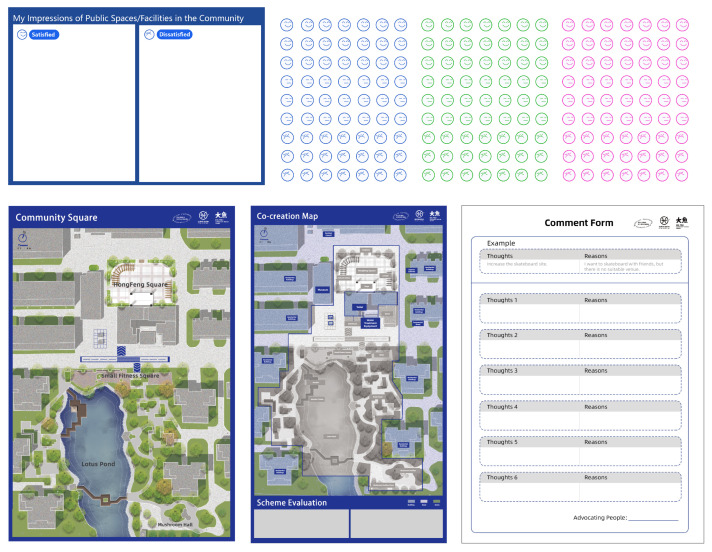
Co-creation workshop toolkits.

We divided the residents into three mixed-age groups and hired professional facilitators to assist the participants in completing the follow-up activities. We designed toolkits for each phase of the co-creation process to ensure the effectiveness of the collaboration, enhance visualization of communications and provide a practical space for the participants to complete the tasks. Some of the toolkits can be found below (Figure 3).

**Figure 4:**
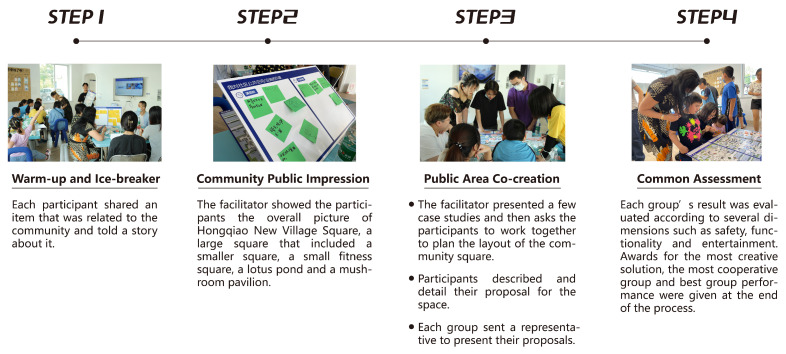
Co-creation workshop flow.

During the co-creation workshop (Figure 4), we noted that residents of all ages voiced their needs and expressed their opinions about the ideal uses of the square. Although the discussions featured differences and heated debates, the dual effects of the toolkit and the facilitator were that participants were able to reach a consensus and generate many creative and rich solutions. The process is shown in the figure below. After the participatory research and co-creation workshops, we conducted in-depth interviews with the participants.

#### 3.2.3. Community Square Exhibition

**Figure 5:**
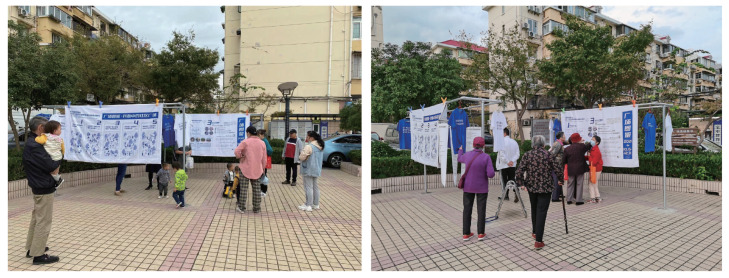
Community square exhibition.

**Figure 6:**
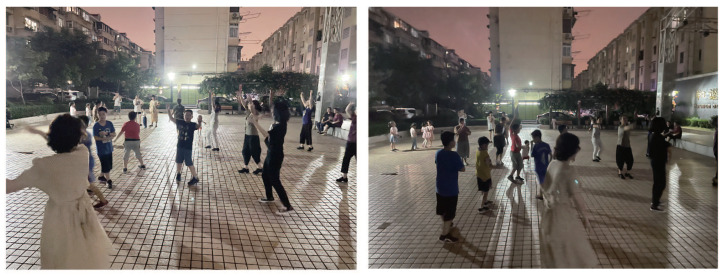
Community residents of all ages dancing together in a public square.

Furthermore, to enable the community to fully understand each other’s ideas, we visualized the prelude information from the pre-participatory research and the conceptual plan output from the co-creation workshop, and we selected the local community public square as the exhibition venue. Finally, we asked the residents to fill in the same questionnaire once again (Figure 5). In the follow-up survey, we found that there was a decrease in the number of conflicts in the square during the night when young people danced together with older residents (Figure 6).

In addition, many of the interviewees indicated their belief that the co-creation approach facilitated communication and led to a deeper understanding of other groups’ ideas, resulting in a gradual reduction in intergenerational prejudice. Moreover, the combination of integration and divergent viewpoints allowed for a more democratic and fair exchange of ideas overall, which led to deeper understanding between the various groups and the creation of new experiences [42].

## 4. Findings and Conclusions from the Project

### 4.1. The Essential Causes of Public Space Conflicts in the Hongqiao Community

Once the participatory community research workshop had concluded, we collected and analyzed the residents’ demands and summarized the essential causes of spatial conflicts in the Hongqiao community’s public square.

(1) More serious attitudes and negative feelings among community residents due to intensified conflicts in public spaces.

Furthermore, we concluded from the quantitative data of the participatory research that there was more interpersonal tension among the local residents (pre-intervention). A certain number of participants showed an “average” or even “poor” relationship with other local community residents. Data on another “hostility” factor (which responds to a subject’s tendency to argue with others and not control their temper) was also high. Then, we took a qualitative research approach of user interviews to further understand residents’ perceptions of various generational groups. When talking about conflicts and problems encountered in public spaces, participants of different age groups became emotional and showed disrespect to other generational groups (some of them) in their speech. This reflected that the relationship between the local residents was not harmonious.

(2) The surface of phenomena exacerbates groups’ one-sided perceptions of each other; the stereotypical labeling of groups is evident during phenomena.

Conflicting desires in the square are reflected in the lively children shuttling around the squares on their bicycles and in play on foot; there are also children running back and forth on stage. At this point, the square dance leaders chase the children away rather rudely, and this pattern is repeated many times. Over time, the actual image seen by other groups in the square is that of older people rudely driving away children, creating a bad impression of some of the older groups, and of older people in general.

(3) Because of the public nature of the square, each group applies their own biases to explain their lack of empathy for the other groups.

In our research, we found that both groups stated that the square belongs to everyone, and everyone has the right to use it. Each group sees the problem from its own perspective, but although no one’s starting point is not wrong, conflict between the groups becomes more and more intense as time goes on. The following are excerpts from the views of some groups.

“We spent an hour and a half dancing with the children, running around unregulated, in the midst of our bike runs—how are we going to hurt them? This has happened before, so who is responsible for this? A square dance should be in the middle of the square, with left and right symmetry.” (O2)

“We were playing in the square, as soon as we got close to them the dancers, we heard the grandmother yelling at us from the side, not even letting us play nicely, and we were out with the kids for such a long time, so why should our freedom be restricted?” (C5)

“Children themselves are running around, the square is also to give them a free space to move together, there should not be too much restriction. The dancers should talk to the children with a little better attitude; they forget, they are also in the community. But the dancers’ attitude is very bad, and sometimes they fight, which we are not used to seeing.” (M6)

(4) Older people and children lack recognition and respect. Neither group has a platform to express themselves. In addition, the younger groups have no sense of belonging.

In addition to the square dancing problem, we found that the older groups complained about the lack of activity and resting areas, that they had no activity center, and that they were “wandering and exiled”; in reality, the community is equipped with public facilities such as gazebos and resting promenades, and the older groups can be seen resting and chatting throughout the community. So, what are the reasons for these complaints?

In conclusion, according to the study, we found that negative intergroup attitudes have a greater impact on people’s mental health after reaching a particular peak. This leads to children becoming more and more inattentive to the elderly and older groups becoming more and more lonely and out-of-touch, etc. Furthermore, the discrepancy between the older community members’ complaints and the available facilities can be explained by their desire to be valued, cared for and respected, rather than marginalized by other community groups.

In addition, children’s rich imaginations and creativity should be explored and valued in a community. Based on our observations and interviews, young people were unable to find a place in the community where they could relate to each other.

### 4.2. Quantitative Analysis of Intergenerational Attitudinal Perceptions

To quantify the intergenerational relationships among community residents, the effectiveness of the co-creation approach was verified by comparing pre- and post-co-creation interventions using the Intergenerational Attitudes Scale (Table 1).

**Figure 7:**
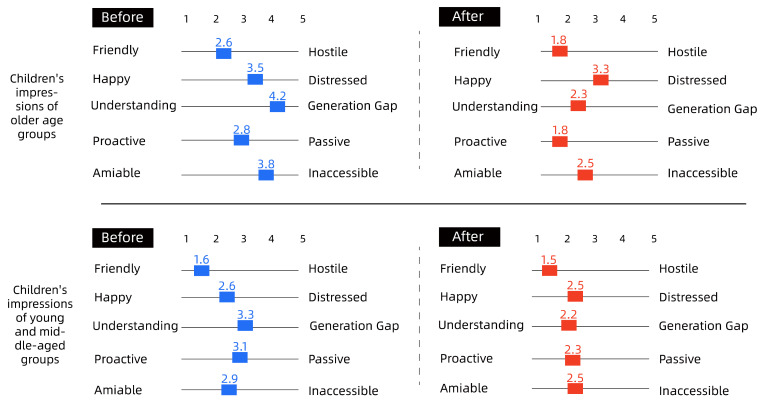
Children’s impressions of different ages. The children’s impressions of the different groups changed significantly and positively after the collaborative co-creation.

**Figure 8:**
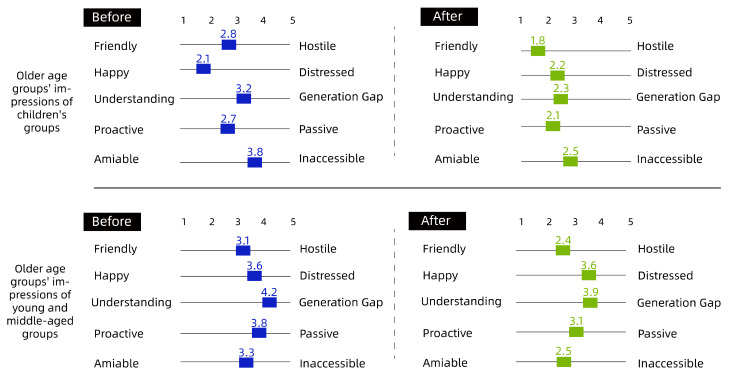
Old age groups’ impressions of different ages. The change in attitudes of the older age group toward the other groups was not significant.

**Figure 9:**
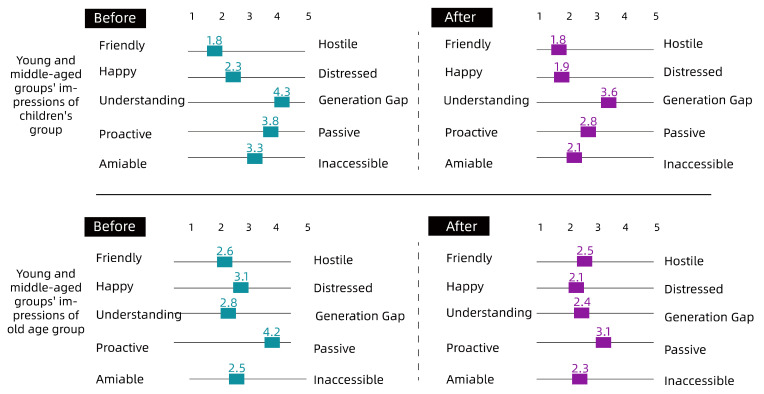
Young and Middle-Age Groups’ Impressions of Different Ages. After the intervention, the perceived impressions of the middle-aged and young adult groups were more proactive and approachable than before, while the attitudes toward children were perceived as more friendly and more welcoming than those of the older group.

The details are shown in Figure 7, Figure 8 and Figure 9.

Overall, as shown in Figure 7, Figure 8 and Figure 9, there was a degree of improvement in perceptions of each other through the collaborative intervention, especially in the ‘Understanding-Generation Gap’, ‘Proactive–Passive’, ‘Amiable–Inaccessible’ scales; for example, children’s perceptions of the older group as generational and inaccessible before the intervention decreased significantly, from 4.2 to 2.3, 2.8 to 1.8 and 3.8 to 2.5, respectively. The attitude of children to young and middle-aged people had also obviously changed; for example, the indicator of ‘Understanding-Generation Gap’ went from 3.2 to 2.2, and the indicator of ‘Proactive–Inaccessible’ went from 3.1 to 2.3; the change in the older group’s attitude toward other groups was relatively small, but during the interview, many older people said they were better able to understand the children after hearing their perspectives and desires. Regarding the insignificant difference in the impressions of the middle-aged group, it could be seen from the graph that the overall impressions of the middle-aged group before the intervention were more positive toward the older group and the child group. As such, the difference between before and after the intervention was small, and the changes in values were mainly concentrated in ‘Understanding-Generation Gap’ and ‘Proactive–Passive’. The authors then conducted a study on middle-aged participants. The summary of the research results showed that the middle-aged group’s own cognitive judgment is more solid, and that the results do not change significantly. For more information on the scales, please refer to the Supplementary Materials, Supplementary S1 (Community Needs) and Supplementary S2 (Survey).

Additionally, many of the participants in the interviews indicated that the co-creation approach facilitated communication and co-creation among the participants, leading to a deeper understanding of other groups’ perspectives and a gradual reduction in intergenerational prejudice. It is the empathy of local residents and the positive change in their perception of each other that has led to a gradual reduction in conflicts in the public spaces of the community. The enthusiasm and willingness of the people to participate in activities has also increased, which is a favorable sign of a healthy community ecology. Moreover, the combination of integration and divergent perspectives allowed for a more democratic and fair exchange of ideas among participants, leading to a deeper understanding among groups and the creation of new experience.

### 4.3. Co-Creation Methodology Conclusions

At the end of the practice project, we reviewed the methods and activities within the design process to summarize the strengths and weaknesses of the activities and where they can be improved.

#### 4.3.1. Community-Based Participatory Research

The ‘Truth or Dare’ form of community participation is a new experiment. The theoretical basis for this participatory research comes from sociologist Randall Collins, who theorizes that the underlying framework of social structure is the ‘ritual chain of interaction,’ which emphasizes interactions that are structured in time through dynamic contact between individuals in specific contexts to form an activity structure. Collins posits that the increasing number of groups participating in social encounters and the extension of the natural space from which these encounters emerge creates a macro-social framework. Therefore, with the help of the community exhibit, the team proposed the concept of ‘community day’ and tried to create the first ‘encounter’ between residents at scale through thematic activities close to community life, leading to a ‘community interaction chain’ that the team developed as the prototype of a social structure.

Although research projects such as ours have made some progress, there are still many problems. For example, (1) the universality of the activity format was weak, and the participants of the weekend community day were mainly children and older people, while the participation of other groups was relatively low. This is because young and middle-aged people did not have a high demand for public space in the community, the community was relatively closed, and the team had not undertaken extensive publicity. (2) The reward mechanism was not appropriate. The team originally wanted to increase the motivation of participants through rewards that differed according to the number of answers. As mentioned above, although many residents were attracted, most of them were children and some of them did not answer the questions seriously enough to earn additional prizes, which made the results invalid. The effectiveness of rewards should be carefully considered when organizing such activities to avoid counterproductive effects. (3) In addition to the reward mechanism, some of the questionnaires were obscure and difficult to understand, making them incomprehensible to some residents and requiring special explanation by the researcher to complete. Therefore, the themes and content of the activities for all ages should be universal to reduce the group’s frustration and trial-and-error rate.

#### 4.3.2. Community Co-Creation Workshop

The problems that emerged from the workshops were mainly focused on pre-recruitment and the dynamic development process. (1) The participation of older adult groups was low, which was mainly because they did not receive the relevant information through common dissemination channels, and they did not think they were suitable for this kind of activity and were worried about feeling left out. (2) During the activity, the conflict between the groups might have blocked some of the process. (3) The children’s groups lacked team consciousness, had poor execution when co-creating and did not understand some of the processes.

The team explored each of these situations. The older adult group was active in the tea party and the participatory research session, but why did they resist the invitation to collaborate with other groups in the design of the new system? Through in-depth discussions, we found that this was due to their sense of marginalization, the fear of being disconnected from society and the fear of not being able to play a role, and therefore, they refused to participate. A topic for future consideration is how to improve the self-confidence of this group and give them a sense of respect.

Additionally, there were conflicts and disagreements throughout the workshop. If such conflict points are properly controlled, they can facilitate communication and improve mutual understanding; if poorly controlled, they can intensify and hinder progress. The team encountered the latter in practice when the two sides disagreed over some features of the public square, and the younger participants dropped out of the workshop in deference to their elders. To avoid such situations, the team suggested that a democratic vote can be taken by secret ballot, and that if there is stagnation, a ‘filing’ mechanism can be used to suspend the current conflict and move forward with the next action.

Finally, developing the children’s sense of teamwork requires experienced facilitators and ground rules that fit a younger mindset. Once these are established, a detailed ‘vision’ and goals can be created, and children can be informed that this will only be accomplished through cooperation among participants, thus keeping their motivation high.

## 5. Discussion

Through our research, we verified that even in the face of more serious and sensitive issues and difficulties, after spending time together and participating in co-creation activities, each age group reported mutual understanding and an improved relationship with other community residents. In follow-up surveys, the number of conflicts in public space had decreased. Residents also indicated that when they encountered similar disputes, they would first understand the specific situation and then deal with it through negotiation. This shows that through co-creation interventions, people are empowered to feel respected and gradually move from being served to being active contributors to the community. This ultimately benefits people’s psychological well-being and, moreover, enhances the group’s social happiness.

We summarized the positive friendship relationship of the intergenerational group, the driving factors and constraints in the process of organizational synergy and how they are related to each other. In addition, we established the co-creation model by combining our results with previous academic research and practice.

### 5.1. Influencing Factors and Constraints in Promoting Intergenerational Integration

Through a case practice, we have verified that the intervention of co-creation in community design can effectively improve group relations in public spaces. Therefore, combined with related studies aimed at enhancing social well-being, we posit that the drivers of the co-creative design approach under I-I are the effectiveness of intergenerational information communication, the visibility of I-G interaction, the cultural applicability of themes and the accessibility of the design tools, including the intergenerational stereotypes (social roles) of I-Gs and I-Gs’ stance on co-participation. These drivers and barriers are described below.

#### 5.1.1. Influencing Factors

- Cultural Applicability of Theme Settings

A universal theme helps individuals integrate more easily into a communicative situation. Given the often high levels of diversity within a community (e.g., varying cultural backgrounds, ages and jobs), it is important to consider whether community members’ interactions align with people’s general perceptions of the community as a whole. In our study, members of the young and middle-aged groups, especially the youths, participated more actively in the co-creation activities. This active participation and the fact that these two groups interacted more frequently with each other than with the older group resulted in many creative conceptual solutions. The older groups participated relatively passively in their activities, meaning that the cultural applicability of these themes had far-reaching significance [43]. We can choose universal topics such as how to view community public spaces, how to control littering, home study during the epidemic etc., which can enhance the participation of the elderly group and combine their rich experience to produce effective solutions [44,45].

- Information Effectiveness of I-Cs

Information effectiveness plays a decisive role in facilitating I-Cs, and the effectiveness of communication depends on the output. This statement refers not only to the expression of natural language (communication) but also to the use of non-verbal and other multidimensional senses, such as the body, to enrich information output channels, which is a process that also relates to the workings of the human brain. In addition, input is the reception of information, and I-Gs may experience communication difficulties because of intergenerational cognitive barriers, language communication differences and other issues. As such, it is important to avoid asking participants to engage in overly complicated activities and to allow individuals of all ages to participate [46,47].

At the same time, designers should focus on bridging intergenerational gaps in communication and easing pressure on participants to interact with one another. During the project, some of the older participants, who did not communicate with the other participants, were unfamiliar with the processes and instructions, slowing down the progress of some groups. This eventuality acted as a reminder to the designers to manage the process using rational measures. Examples include the use of more pictures, with a small amount of text and a guide to explain the details of the process. Alternatively, they can integrate the research questions and tasks in the storyline to enhance the effectiveness of the message in a narrative way [48,49,50].

- Visibility of I-G Interactions

Visibility is the visual presentation of information. The activities of this pilot project were presented in table form, and the participants were asked to discuss ideas and elaborate on concepts. Although this method proved useful, there was room for further improvement in the way that the participants interacted with the information. We suggest that in future studies, the number and complexity of the tables should be controlled, and the design of each stage should be as concise and precise as possible so that participants can easily understand the purpose of the various tasks and the steps they need to take in a relatively short space of time. We also suggest that future studies pay attention to the appropriate distribution of text and images and use appropriate forms such as cards and stickers to effectively present information, enhance the presentation of conceptual information, avoid cognitive frustration and motivate group participation.

- Effective Use of GIS in Community Public Health

The strength of GIS lies in presenting the results of analysis (i.e., patterns in the data) in the form of maps that are highly influential as well as visually expressive. These maps combine multiple data in space as a way to answer various questions [51]. Therefore, the application of GIS technology to community health information collection, health status tracking, and policy planning is of great importance [52,53].

Although this technical application for GIS only relied on visual presentation, it also had provided us with a lot of useful information. Therefore, in the subsequent study, we plan to collect a wide range of physical and mental health conditions of community groups and combine them with the community spatial environment to dynamically track community conditions and build a community information map in all aspects. In turn, we will plan and design corresponding activities and propose reasonable measures to improve spatial and intergenerational relationships as well as related policies to address the challenges of the future.

#### 5.1.2. Constraint Factors

- Intergenerational Stereotypes of Groups (Social Roles)

Social relationships and social roles are also included in the determinants of well-being and mental health [54]. The Intergenerational Impression scale shows that the younger community members had a one-sided and stereotypical image of the older group, which indirectly led to the younger group deliberately reducing or even avoiding interactions with the older participants in the experimental activities. During the post-activity interviews (user interviews), the younger participants also mentioned their reluctance to communicate because they felt that older people were “stubborn” and “old-fashioned”. The older participants showed a willingness to communicate with the younger participants, who were perceived to be full of energy and ideas. At the same time, the older participants were concerned that they would be unable to play to their strengths; as a result, they often remained silent, which led to misunderstandings, contradictions and conflicts. As such, we posit that it is vital to find ways to improve intergroup perceptions and promote IR.

- Collaborative Participatory Position of the Group

Effective intercultural dialogue should be based on an egalitarian framework, that is, treating all people equally and avoiding imposing one group’s characteristics on other groups with different cultural backgrounds. At the same time, participants may experience a certain degree of conflict as a result of the influence of diverse elements, including the presence of different types of people. For example, in the case of the co-creation activity, one group of young people reported feeling that older people could be more active in outings and group activities to foster relationships, but many members of the older group reported feeling more comfortable remaining in the community playing chess and chatting with their peers. This conflict led to intense discussions, but no agreement was reached. Our analysis showed that cultural differences stemmed from differences in understanding. We therefore suggest that using empathy and promoting conceptual convergence require experiential interaction that leads to new experiences and understanding [55].

- The Participants’ Collective Consciousness was not Unified

During the activity, we found that each group experienced varying degrees of slowdown. We believe that this situation arose from the disorganization and actions of the participants [56]. Although the participants discussed and co-created on the basis of their common interests, the participants were autonomous in their choice of strategies, which they based on their personal preferences. For example, some participants wanted to dominate the discussion, providing the other participants with fewer opportunities to participate. In addition, the younger participants did not fully understand the rules, which may have affected the development of a collective consciousness. As such, we suggest that when organizing activities involving children, researchers should provide a sound processing system and experienced facilitators to assist children in completing their research, co-creation, and other activities.

### 5.2. Co-Creation Model

**Figure 10:**
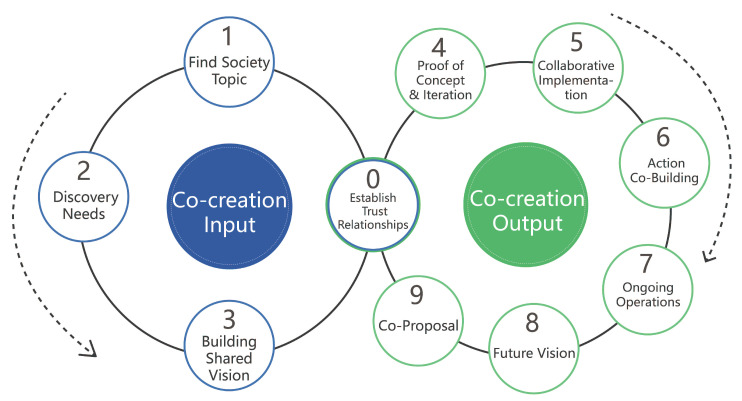
Co-creation method model.

In addition, we have established a theoretical framework for co-creation to promote intergenerational integration in communities. In response to the conflicts encountered by groups in public settings, theoretical approaches are used to uncover the core issues and eventually organize the masses to find a solution. The details are shown below (Figure 10).

#### 5.2.1. Input of the Co-creation Method Model

As shown in Figure 10, the input and output components intersect to cultivate trusting relationships that are interwoven into the overall methodology, as promoting intergenerational integration in the community is centered on building trusting relationships among the community’s residents [57]. Trusting relationships are divided into an outer core and an inner core. The outer core refers to the cooperation and camaraderie between the researcher (designers, stakeholders) and the residents, and the inner core reflects the mutual understanding, recognition and respect between community residents of all ages. The creation and establishment of trusting relationships is a part of the overall process of community design. As such, the foundation for co-creation is laid through the creation of relationships between the inner core and the outer core.

Secondly, the model of ‘everyone initiates’ informs our methodological approach to exploring problems that arise, using the theoretical framework of this co-creation approach. This method encourages community residents of all ages to act as the callers and initiators of community action, to raise questions and to organize discussions on community issues with the aim of jointly finding appropriate solutions. The construction of the ‘everyone initiates’ model should be based on the benefits of community relations as well as the development of systematic and comprehensive guidelines for community members to organize discussions amongst themselves.

The researchers use GIS technology to analyze and build community maps in a comprehensive manner to gain insight into the community environment and the life trajectory of local residents. Once the community identifies its problems, the researcher’s goal is to identify the needs of the community and reach a consensus on establishing a common vision. To explore the needs of the community, we suggest engaging community members in discussions using the internal participatory research method. In the process of endogenous research, we can combine the local properties of the community and the real activities of the residents by holding an open day for the community. This process alters the traditional roles of the interviewer and interviewees by using alternative interview formats, for example interactive boards, booths and exhibitions, and allowing neighbors to participate in the conversation. Researchers may also consider using incentive mechanisms to increase participation.

#### 5.2.2. Output of the Co-Creation Method Model

For the output aspect of the co-creation method, community residents’ joint proposals and co-creation are important elements of the initial research phase. Depending on the issue that needs to be addressed in the community, the content and forms of co-creation activities will vary. Combining our preliminary case collection and practical research, we suggest that the elements for conducting intergenerational co-creation sessions should include a targeted participant screening mechanism, a universal multidimensional experience process, experienced facilitators and a professional toolkit for information visualization.

The community residents iterate this output concept after resolving the community co-creation issues. Resident validation is broken down into internal iterations and peripheral open proposals. Internal validation will enable groups to evaluate their impressions of the other groups after the implementation of co-creation. Typically, co-creation workshops are conducted in groups, and at the end of the workshop, audience members are permitted to discuss and share ideas with one another. This step requires evaluation criteria that can be established either by the research team designing a basic evaluation template based on topic or by the residents themselves, who would be asked to develop their own evaluation dimensions and degrees.

The community implementers’ collaboration is also vital in motivating the participants. As the community project progresses, the research team, stakeholders and community participants will carry out the initial research, identify and conceptualize the problem and go through the interactive process to produce a plan that meets the needs of the community and has the approval of the audience. The various participants will establish mutual understanding and develop an awareness of the community and its needs. Although the collaborative process is extremely useful, there are also many problems associated with it that require researchers to establish strategies to monitor the implementation of the project in real time and to provide dynamic feedback and coordination when problems arise. An effective way to address these concerns is to build a self-organizing mechanism that involves stakeholders in the process of providing real-time supervision. In addition, researchers can use existing communication platforms to form an interoperable network of neighborhood committees, residents, research teams and builders to provide immediate feedback on the project.

Academics and practitioners are exploring sustainable operations and visions for the future [58,59]. At present, individuals involved in community creation and design in many practice projects have accumulated much experience and have established a variety of rules for the early incubation and mid-term establishment stages. However, the long-term management and maintenance of the latter stages is still in an experimental state, because design teams are mostly removed from their projects. This feature often results in improper management or a complete lack of management, rendering any results wasted. We therefore suggest establishing an autonomous group led by a research team that brings together the community committee and other stakeholders to discuss the basic operations of the project. In other words, we suggest the establishment of a targeted program that considers the attributes of the output, enhances the sustainable endogenous power of the community and emphasizes the important role of each group to establish a future vision and promote the needs of the community through its operation.

## 6. Conclusions

In the face of positive developments in future cities and communities, society is increasingly concerned about people’s mental health, intergenerational interactions, and other soft-level relationships in public environments. This study delves into the positive transformation of intergenerational relationships in community contexts after the intervention of design forces. Through collaborative co-creation, the role of communities is gradually transformed to enhance their well-being and create a positive public health and all-age friendly atmosphere. It also lays the foundation for sustainable community operation and later maintenance.

Throughout history, strong intergenerational relationships have been essential for human survival and socialization [60,61]. Using practical examples, this article demonstrates that co-creative interventions in social innovation practices can help promote mutual understanding and alleviate stereotyping across all age groups. We assessed the effectiveness of the co-creation methods multidimensionally using quantitative data, qualitative interviews and a comparison of project outputs. We developed a theoretical model of co-creative design approach for promoting intergenerational integration and creating a healthy environment for positive communities. Meanwhile, the drivers and constraints of the co-creation methods were summarized in practice, leading to the development of tailored design strategies for the application of these methods in future community scenarios. We also highlighted the importance of GIS in the future of community health.

In addition, we expanded the pool of participatory designs and workshops to develop research methods applicable to promoting intergenerational communication and cooperation. Through specific practice, it proved that high-quality intergenerational interactions (communication, collaboration, etc.) improve intergenerational relationships to a certain extent and lead to a healthy and positive psychological state.

This project has certain limitations, such as scalability. For example, considering the cognitive level of the participants and the feasibility of the project, we chose a first-tier Chinese city (Shanghai) as the target for the pilot study because of its cultural diversity and strong sense of community belonging, which are the two factors that guaranteed audience participation in the workshops. Furthermore, while also helpful in allowing us to intervene using a co-creation approach that led to relatively smooth and productive collaboration between groups, these features lacked universality. This research was conducted using a combination of theory and practice; it has important implications for organized group co-creation in the community. It is hoped that the results will be expanded and applied to a wider range of contexts to explore a wider range of development possibilities. We also hope that this project will result in the harmonious coexistence of older people with other generations in a variety of locations.

## Figures and Tables

**Table 1:** Intergenerational Attitudes Scale.

Age				Sex		
	Strongly disagree	Disagree	Neutral	Agree	Strongly Agree	
	5	4	3	2	1	
Friendly						Hostile
Happy						Distressed
Understanding						Generation Gap
Proactive						Passive
Amiable						Inaccessible

## Data Availability

To protect the privacy of participants, the questionnaire data will not be disclosed to the public. If necessary, you can contact the corresponding author.

## Supplementary Materials

The following supporting information can be downloaded at: https://www.mdpi.com/article/10.3390/ijerph191912908/s1, Supplementary S1: Demand of community; Supplementary S2: Survey.

## References

[1] Armstrong L., Bailey J., Julier G., Kimbell L. (2014). Social Design Futures: HEI Research and the AHRC.

[2] Alan H.-Y., Batty C. (2011). Evaluating the contribution of intergenerational practice to achieving social cohesion. Promoting Social Cohesionimplications for Policy and Evaluation.

[3] Ahrens L. (2020). Corona Krise: Wir bBrauchen jetzt Solidaritat mit der Jungen Generation. Hamburger Morgenpost. https://www.mopo.de/hamburg/meinung/corona-krise-wir-brauchen-jetzt-solidaritaet-mit-der-jungen-generation--36748854.

[4] Willumsen E., Ødegård A., Sirnes T. (2019). Co-creation and integration when improving residential care for the elderly. Int. J. Integr. Care.

[5] Li Q., Yan L. (2018). Understanding older adults’ post-adoption usage behavior and perceptions of mobile technology. Int. J. Des..

[6] Riley M.W., Riley Jr J.W. (2000). Age integration: Conceptual and historical background. Gerontologist.

[7] Ray D. (2021). Intergenerational Trauma and Cognition. Bachelor’s Thesis.

[8] Ball A., Rivkah C. (2011). More than words–intergenerational participation and mental health. Ment. Health Soc. Incl..

[9] Landstedt E., Almquist Y.B. (2019). Intergenerational patterns of mental health problems: The role of childhood peer status position. BMC Psychiatry.

[10] Murayama Y., Ohba H., Yasunaga M., Nonaka K., Takeuchi R., Nishi M., Sakuma N., Uchida H., Shinkai S., Fujiwara Y. (2015). The effect of intergenerational programs on the mental health of elderly adults. Aging Ment. Health..

[11] Markussen T. (2017). Disentangling ‘the social’ in social design’s engagement with the public realm. CoDesign.

[12] Laclau E., Mouffe C. (2014). Hegemony and Socialist Strategy: Towards a Radical Democratic Politics.

[13] Ongenae F., Duysburgh P., Sulmon N., Verstraete M., Bleumers L., De Zutter S., Verstichel S., Ackaert A., Jacobs A., De Turck F. (2014). An ontology co-design method for the co-creation of a continuous care ontology. Appl. Ontol..

[14] Chen Z. (2018). Research on the Design of Community Elderly Services Based on Intergenerational Inclusion. Ind. Des..

[15] https://onlinedegrees.und.edu/blog/what-is-community-mental-health/.

[16] Thornicroft G., Deb T., Henderson C. (2016). Community mental health care worldwide: Current status and further developments. World Psychiatry.

[17] Simmonds S., Coid J., Joseph P., Marriott S., Tyrer P. (2001). Community mental health team management in severe mental illness: A systematic review. Br. J. Psychiatry.

[18] Sanders E.B.N. (2002). From user-centered to participatory design approaches. Design and the Social Sciences.

[19] Hagestad G.O., Uhlenberg P. (2005). The Social Separation of Old and Young: A Root of Ageism. J. Soc. Issues.

[20] Tsekleves E., Bingley A., Escalante M.A.L., Gradinar A. (2020). Engaging people with dementia in designing playful and creative practices: Co-design or co-creation?. Dementia.

[21] Ibinarriaga D.H., Martin B. (2021). Critical Co-Design and Agency of the Real. Des. Cult..

[22] Nthubu B., Perez D., Richards D., Cruickshank L. (2022). Navigating Complexity through Co-design: Visualising, Understanding and Activating Entrepreneurial Ecosystems. Des. J..

[23] Calvo M., De Rosa. A. (2017). Design for social sustainability. A reflection on the role of the physical realm in facilitating community co-design. Des. J..

[24] Biro-Hannah E. (2021). Community adult mental health: Mitigating the impact of COVID-19 through online art therapy. Int. J. Art Ther..

[25] Aguirre C., Astbury J., Gridley H., Sharples J. (2011). Benefits of Group Singing for Community Mental Health and Wellbeing.

[26] Fiorillo A., Gorwood P. (2020). The consequences of the COVID-19 pandemic on mental health and implications for clinical practice. Eur. Psychiatry.

[27] Lou V.W.Q., Dai A.A.N. (2017). A Review of Nonfamilial Intergenerational Programs on Changing Age Stereotypes and Well-Being in East Asia. J. Intergenerational Relatsh..

[28] Sanders E.B.-N., Stappers P.J. (2008). Co-creation and the new landscapes of design. CoDesign.

[29] Carpenter J., Christina H., Ben S. (2021). Co-Creation as an agonistic practice in the favela of Santa Marta, Rio de Janeiro. Urban Stud..

[30] Botero A., Hyysalo S. (2013). Ageing together: Steps towards evolutionary co-design in everyday practices. CoDesign.

[31] Polizzi K.G. (2003). Assessing attitudes toward the elderly: Polizzi’s refined version of the aging semantic differential. Educ. Gerontol..

[32] Vanderbeck R.M. (2007). Intergenerational geographies: Age relations, segregation and re-engagements. Geogr. Compass.

[33] Kelly A.C., Zuroff D.C., Leybman M.J., Gilbert P. (2012). Social safeness, received social support, and maladjustment: Testing a tripartite model of affect regulation. Cogn. Ther. Res..

[34] Binyamin G., Friedman A., Carmeli A. (2018). Reciprocal care in hierarchical exchange: Implications for psychological safety and innovative behaviors at work. Psychol. Aesthet. Creat. Arts.

[35] Natalie R., Jarjue E., Kacorri H., Hara K. The efficacy of collaborative authoring of video scene descriptions. Proceedings of the 23rd International ACM SIGACCESS Conference on Computers and Accessibility.

[36] Molina-Betancur J.C., Agudelo-Suárez A.A., Martínez-Herrera E. (2021). Grassroots innovation practices for social transformation of the health and well-being in a self-built settlement in Medellín-Colombia. Health Soc. Care Community.

[37] Kraff H. (2020). A Critical Exploration of Agonistic Participatory Design. Des. J..

[38] Lukat J., Margraf J., Lutz R., van der Veld W.M., Becker E.S. (2016). Psychometric properties of the Positive Mental Health Scale (PMH-scale). BMC Psychol..

[39] https://wenku.baidu.com/view/1752415fdf3383c4bb4cf7ec4afe04a1b171b073.html.

[40] Church R.L. (2002). Geographical information systems and location science. Comput. Oper. Res..

[41] Kvamme K.L. (1999). Recent directions and developments in geographical information systems. J. Archaeol. Res..

[42] Seedsman T. (2017). Building a humane society for older people: Compassionate policy making for integration, participation, and positive ageing within a framework of intergenerational solidarity. J. Intergenerational Relatsh..

[43] Ma E.W., Jääskeläinen V., Ruokonen I., Karlsson L., Ruismäki H. (2017). Steps Together: Children’s Experiences of Participation in Club Activities with the Elderly. J. Intergenerational Relatsh..

[44] Talmage C.A., Baker A.L., Guest M.A., Knopf R.C. (2020). Responding to social isolation among older adults through lifelong learning: Lessons and questions during COVID-19. Local Dev. Soc..

[45] Kruijf J.V., Verbrugge L., Schröter B., Haan R.-J.D., Arevalo J.C., Fliervoet J., Henze J., Albert C. (2022). Knowledge co-production and researcher roles in transdisciplinary environmental management projects. Sustain. Dev..

[46] Cartestenses L., Mason S.E., Caldwell E.C. (1982). Children’s attitudes toward the elderly: An international techniques for a change. Educ. Gerontol..

[47] Kim C., Nam K.Y. (2021). Policy Puzzle Game: Making policy ideas feasible and acceptable in policy co-design. CoDesign.

[48] Birch J., Parnell R., Patsarika M., Šorn M. (2017). Creativity, play and transgression: Children transforming spatial design. CoDesign.

[49] Roig A., de Sá F.P., Cornelio G.S. (2018). Future Story Chasers: An experience with co-creation of fiction in the classroom through a collaborative storytelling game. Catalan-J. Commun. Cult. Stud..

[50] Wu J., Zhang L., Qian Z.C., Li R. (2019). Applying Storytelling Method into the Flow of User Experience Design to Innovate with Serendipity. A case study on AIDS detection service design among college students. Des. J..

[51] Kistemann T., Friederike D., Jürgen S. (2002). New perspectives on the use of Geographical Information Systems (GIS) in environmental health sciences. Int. J. Hyg. Environ. Health.

[52] Pine J.C., Diaz J.H. (2000). Environmental health screening with GIS: Creating a community environmental health profile. J. Environ. Health.

[53] Plescia M., Koontz S., Laurent S. (2001). Community assessment in a vertically integrated health care system. Am. J. Public Health.

[54] Keyes C.L.M., Mary B.W. (2003). Dimensions of well-being and mental health in adulthood. Well-Being: Positive Development across the Life Course.

[55] Lam B., Phillips M., Kelemen M., Zamenopoulos T., Moffat S., de Sousa S. (2018). Design and Creative Methods as a Practice of Liminality in Community-Academic Research Projects. Des. J..

[56] Olson M. (2012). The Logic of Collective Action [1965]. Contemporary Sociological Theory.

[57] Cohavi O., Levy-Tzedek S. (2022). Young and old users prefer immersive virtual reality over a social robot for short-term cognitive training. Int. J. Hum. Comput. Stud..

[58] Bratteteig T., Wagner I. What is a participatory design result? In Proceedings of the 14th Participatory Design Conference, Aarhus, Denmark, 15–19 August 2016; Volume 1.

[59] Bengtson V.L., Black K.D. (1973). Intergenerational Relations and Continuities in Socialization. Life-Span Developmental Psychology.

[60] Marston H.R., Niles-Yokum K., Silva P.A. (2021). A Commentary on Blue Zones^®^: A critical review of age-friendly environments in the 21st century and beyond. Int. J. Environ. Res. Public Health.

[61] Ko H., Jung S.J. (2021). Association of social frailty with physical health, cognitive function, psychological health, and life satisfaction in community-dwelling older Koreans. Int. J. Environ. Res. Public Health.

